# Supplementary material for: Pavement Quality Index Rating Strategy Using Fracture Energy Analysis for Implementing Smart Road Infrastructure
Source: Sensors (Basel). 2021 Jun 20;21(12):4231. doi: 10.3390/s21124231 (PMC8235173; doi:10.3390/s21124231)
Supplement: Supplementary file 1 [file sensors-21-04231-s001.zip › sensors-1165999-supplementary.pdf]

APPENDIX I

GRIFFITH ENERGY RELEASE FOR PAVEMENT FAILURE CONSIDERING TEMPERATURE AS ENVIRONMENTAL INPUT PARAMETERS

Time	Temperature 1	Temperature 2	Temperature 3	**GT (T1)**	**GT(T2)**	**GT(T3)**	**E (T1)**	**E(T2)**	**E(T3)**
10	23,87	24,06	23,44	1,751691	1,710227	1,847944	398204,4	395738,2	403842,5
20	24,62	24,75	24,25	1,59182	1,565145	1,669416	388558,1	386910	393287,4
30	25,06	25	24,69	1,502769	1,514706	1,577419	383008,1	383760,3	387669,8
40	24,94	24,87	24,69	1,526708	1,540793	1,577419	384513,9	385394,9	387669,8
50	25,12	25,06	24,87	1,490896	1,502769	1,540793	382257,5	383008,1	385394,9
60	25,37	25,19	25,19	1,442125	1,477127	1,477127	379145,6	381383,6	381383,6
70	25,75	25,62	25,5	1,370139	1,394475	1,417207	374463,9	376059	377537,4
80	26,06	25,94	25,81	1,31332	1,335112	1,359008	370687,6	372144,8	373730
90	26,12	25,94	25,94	1,302519	1,335112	1,335112	369961	372144,8	372144,8
100	26,19	26	26	1,289999	1,324184	1,324184	369115,3	371415,5	371415,5
110	26,37	26,25	26,19	1,2582	1,279336	1,289999	366949,2	368391,8	369115,3
120	26,69	26,56	26,5	1,203068	1,22525	1,235587	363129,9	364676,7	365392,8

130	26,94	26,81	26,75	1,161233	1,182852	1,192929	360173,7	361707,9	362418,2
140	27,12	26,75	26,87	1,131777	1,192929	1,172838	358060,2	362418,2	360999
150	27,12	26,75	26,87	1,131777	1,192929	1,172838	358060,2	362418,2	360999
160	27,12	26,69	26,94	1,131777	1,203068	1,161233	358060,2	363129,9	360173,7
170	27,06	26,62	26,87	1,141534	1,214976	1,172838	358763,3	363962	360999
180	27,25	26,94	27,06	1,110848	1,161233	1,141534	356541,4	360173,7	358763,3
190	27,5	27,06	27,25	1,071404	1,141534	1,110848	353638,9	358763,3	356541,4
201	27,75	27,12	27,44	1,03301	1,131777	1,080775	350760	358060,2	354333,3
211	28,19	27,56	27,81	0,967949	1,062094	1,02395	345749,8	352945,8	350072,5
221	28,62	27,75	28,19	0,907405	1,03301	0,967949	340922,8	350760	345749,8
231	28,56	27,56	28,12	0,915675	1,062094	0,978088	341592,2	352945,8	346542,1
241	28,81	27,87	28,37	0,881591	1,01495	0,942244	338811,4	349386,4	343720,9
251	28,44	27,56	28,19	0,932388	1,062094	0,967949	342935,1	352945,8	345749,8
261	27,62	26,94	27,62	1,052845	1,161233	1,052845	352254,1	360173,7	352254,1
271	27,12	26,69	27,12	1,131777	1,203068	1,131777	358060,2	363129,9	358060,2
281	26,81	26,31	26,81	1,182852	1,268736	1,182852	361707,9	367669,8	361707,9
291	26,37	25,94	26,37	1,2582	1,335112	1,2582	366949,2	372144,8	366949,2
301	26,25	25,94	26,19	1,279336	1,335112	1,289999	368391,8	372144,8	369115,3

311	26,31	26,06	26,25	1,268736	1,31332	1,279336	367669,8	370687,6	368391,8
321	26,75	26,25	26,62	1,192929	1,279336	1,214976	362418,2	368391,8	363962
331	27,25	26,31	27,19	1,110848	1,268736	1,120472	356541,4	367669,8	357241,6
341	27,62	26,5	27,5	1,052845	1,235587	1,071404	352254,1	365392,8	353638,9
351	27,75	26,56	27,62	1,03301	1,22525	1,052845	350760	364676,7	352254,1
361	27,87	26,69	27,81	1,01495	1,203068	1,02395	349386,4	363129,9	350072,5
372	27,87	26,69	27,81	1,01495	1,203068	1,02395	349386,4	363129,9	350072,5
382	27,56	26,37	27,56	1,062094	1,2582	1,062094	352945,8	366949,2	352945,8
392	27,44	26,06	27,37	1,080775	1,31332	1,091783	354333,3	370687,6	355145,2
402	27,19	25,81	27,12	1,120472	1,359008	1,131777	357241,6	373730	358060,2
412	26,5	25,56	26,62	1,235587	1,405809	1,214976	365392,8	376797,5	363962
422	25,75	25	25,94	1,370139	1,514706	1,335112	374463,9	383760,3	372144,8
432	25,19	24,5	25,37	1,477127	1,616715	1,442125	381383,6	390085,7	379145,6
442	24,81	24,25	25	1,552937	1,669416	1,514706	386151,7	393287,4	383760,3
452	24,56	23,94	24,69	1,604235	1,736339	1,577419	389321,2	397294	387669,8
462	24,37	23,75	24,5	1,643978	1,778216	1,616715	391747,3	399769,8	390085,7
472	24,12	23,44	24,25	1,697269	1,847944	1,669416	394962,6	403842,5	393287,4
482	23,81	23,12	24	1,764921	1,921736	1,72325	398986,3	408090	396515,3

492	23,44	22,75	23,69	1,847944	2,009345	1,791576	403842,5	413057	400554,9
502	23,12	22,37	23,31	1,921736	2,101853	1,8777	408090	418221,2	405562,7
512	22,87	22,06	23,06	1,980664	2,179199	1,935777	411439,5	422481,8	408891,4
522	22,62	21,94	22,81	2,040706	2,209588	1,994973	414816,5	424142,7	412247,5
532	22,31	21,5	22,56	2,116692	2,323129	2,055281	419042,4	430288,8	415631,1
542	21,94	21,19	22,25	2,209588	2,40509	2,131595	424142,7	434672,3	419865,3
553	21,81	21,06	22,06	2,24279	2,439936	2,179199	425949,4	436523,9	422481,8
563	21,75	21	22	2,258211	2,456113	2,194362	426785,8	437381,1	423311,4
573	21,62	20,87	21,87	2,291836	2,491364	2,22743	428603,8	439244,2	425114,6
583	21,25	20,37	21,56	2,389102	2,629474	2,307452	433820,4	446484,1	429445,4
593	21	20,12	21,31	2,456113	2,699997	2,373173	437381,1	450148,7	432970,2
603	20,81	20	21,12	2,507726	2,734188	2,423819	440106,7	451918,4	435668,3
613	20,75	19,94	21,06	2,524147	2,751364	2,439936	440971	452805,8	436523,9
623	20,69	19,81	20,94	2,540625	2,788764	2,472349	441836,9	454734,6	438240
633	20,62	19,75	20,87	2,559922	2,806109	2,491364	442849,3	455627,6	439244,2
643	20,37	19,5	20,69	2,629474	2,878945	2,540625	446484,1	459367,2	441836,9
653	20,06	19,31	20,44	2,717065	2,934895	2,6099	451032,7	462229,9	445463,4
663	19,81	19,12	20,31	2,788764	2,991345	2,646311	454734,6	465110,3	447360,9

673	19,87	19,06	20,25	2,771471	3,009274	2,663205	453843,4	466023,7	448239,4
683	20	19,06	20,12	2,734188	3,009274	2,699997	451918,4	466023,7	450148,7
693	20	19	19,94	2,734188	3,02725	2,751364	451918,4	466938,8	452805,8
703	19,62	18,69	19,75	2,843871	3,120882	2,806109	457568,4	471695,8	455627,6
713	19,19	18,31	19,5	2,97049	3,237309	2,878945	464047	477593,1	459367,2
724	18,81	18,12	19,25	3,08449	3,296171	2,952668	469848,7	480569,3	463137,6
734	18,75	18	19,12	3,102663	3,333561	2,991345	470771,3	482458,5	465110,3
744	18,62	17,87	19	3,142195	3,374247	3,02725	472776,6	484513,6	466938,8
754	18,56	17,81	18,94	3,160512	3,393087	3,045275	473705	485465,1	467855,8
764	18,56	17,81	18,87	3,160512	3,393087	3,066363	473705	485465,1	468927,8
774	18,5	17,75	18,87	3,178874	3,411965	3,066363	474635,3	486418,4	468927,8
784	18,37	17,56	18,75	3,218809	3,471995	3,102663	476657	489449,6	470771,3
794	18,19	17,37	18,56	3,274436	3,532388	3,160512	479470,6	492499,7	473705
804	18	17,25	18,37	3,333561	3,57071	3,218809	482458,5	494435,9	476657
814	17,81	17,06	18,25	3,393087	3,631658	3,255852	485465,1	497517,1	478530,9
824	17,62	16,94	18,12	3,452998	3,670315	3,296171	488490,4	499473	480569,3
834	17,5	16,81	18	3,491028	3,71233	3,333561	490410,8	501600,5	482458,5
844	17,37	16,69	17,87	3,532388	3,751234	3,374247	492499,7	503572,5	484513,6

854	17,19	16,44	17,69	3,589922	3,832635	3,430882	495406,8	507705,7	487373,6
864	16,87	16,19	17,37	3,692921	3,914473	3,532388	500617,5	511872,7	492499,7
874	16,56	15,88	17,12	3,793505	4,016489	3,612376	505717,5	517087,5	496542
884	16,31	15,56	16,87	3,87514	4,122326	3,692921	509868,3	522526,1	500617,5
894	16,12	15,31	16,62	3,93746	4,205319	3,77398	513045,6	526814,8	504726,4
905	15,94	15,19	16,5	3,996701	4,245235	3,813057	516074	528885,9	506710,6
915	15,94	15,19	16,37	3,996701	4,245235	3,855508	516074	528885,9	508869
925	16,06	15,19	16,25	3,957186	4,245235	3,894795	514053,1	528885,9	510869,5
935	16,25	15,25	16,19	3,894795	4,225271	3,914473	510869,5	527849,4	511872,7
945	16,37	15,25	16,06	3,855508	4,225271	3,957186	508869	527849,4	514053,1
955	16,37	15,19	15,94	3,855508	4,245235	3,996701	508869	528885,9	516074
965	16,31	15	15,81	3,87514	4,308522	4,039598	509868,3	532181,8	518272,3
975	16,19	14,94	15,63	3,914473	4,328527	4,099133	511872,7	533226,8	521331,5
985	16	14,75	15,44	3,976934	4,391922	4,162133	515062,6	536549,8	524580,3
995	15,88	14,63	15,31	4,016489	4,43199	4,205319	517087,5	538659,1	526814,8
1005	15,5	14,44	15,25	4,142222	4,495462	4,225271	523552,2	542015,9	527849,4
1015	15,19	14,31	15,25	4,245235	4,538899	4,225271	528885,9	544324,6	527849,4
1025	15,13	14,13	15,13	4,26521	4,59904	4,26521	529924,5	547537,7	529924,5

1035	15,13	14,13	15,06	4,26521	4,59904	4,288527	529924,5	547537,7	531138,8
1045	15,25	14,06	15	4,225271	4,622423	4,308522	527849,4	548792,3	532181,8
1055	15,31	14,06	14,88	4,205319	4,622423	4,348539	526814,8	548792,3	534274
1065	15,31	13,94	14,69	4,205319	4,662496	4,411954	526814,8	550949,8	537603,4
1076	15,25	13,88	14,5	4,225271	4,682526	4,475415	527849,4	552031,7	540953,6
1086	15,19	13,75	14,38	4,245235	4,725901	4,51551	528885,9	554383,1	543080,2
1096	15,06	13,63	14,31	4,288527	4,765908	4,538899	531138,8	556562,6	544324,6
1106	15	13,5	14,19	4,308522	4,809206	4,578995	532181,8	558933,3	546464,6
1116	14,88	13,38	14	4,348539	4,849127	4,642462	534274	561130,7	549870
1126	14,69	13,19	13,88	4,411954	4,912228	4,682526	537603,4	564627,5	552031,7
1136	14,5	13	13,63	4,475415	4,975175	4,765908	540953,6	568146,1	556562,6
1146	14,31	12,88	13,44	4,538899	5,014839	4,829172	544324,6	570379,6	560030,9
1156	14,19	12,63	13,25	4,578995	5,097209	4,892317	546464,6	575061,1	563520,9
1166	14	12,5	13,19	4,642462	5,139884	4,912228	549870	577510,7	564627,5
1176	13,88	12,38	13,06	4,682526	5,179172	4,955315	552031,7	579781	567032,6
1186	13,81	12,25	12,94	4,705885	5,221612	4,995016	553296,6	582250,7	569261,7
1196	13,69	12,25	13	4,745908	5,221612	4,975175	555471,8	582250,7	568146,1
1206	13,5	12,19	13,13	4,809206	5,241154	4,932124	558933,3	583394,1	565736,2

1216	13,25	12,13	13,19	4,892317	5,260667	4,912228	563520,9	584539,7	564627,5
1226	13,13	12,19	13,25	4,932124	5,241154	4,892317	565736,2	583394,1	563520,9
1236	13,31	12,31	13,38	4,872391	5,202041	4,849127	562416,4	581109,5	561130,7
1247	13,69	12,63	13,63	4,745908	5,097209	4,765908	555471,8	575061,1	556562,6
1257	14,13	13,13	13,94	4,59904	4,932124	4,662496	547537,7	565736,2	550949,8
1267	14,5	13,63	14,31	4,475415	4,765908	4,538899	540953,6	556562,6	544324,6
1277	14,81	14,06	14,63	4,371895	4,622423	4,43199	535498,2	548792,3	538659,1
1287	15,13	14,38	14,94	4,26521	4,51551	4,328527	529924,5	543080,2	533226,8
1297	15,44	14,88	15,31	4,162133	4,348539	4,205319	524580,3	534274	526814,8
1307	15,81	15,31	15,69	4,039598	4,205319	4,079271	518272,3	526814,8	520309,8
1317	16,25	15,88	16,19	3,894795	4,016489	3,914473	510869,5	517087,5	511872,7
1327	16,69	16,44	16,62	3,751234	3,832635	3,77398	503572,5	507705,7	504726,4
1337	17,06	16,94	17	3,631658	3,670315	3,650971	497517,1	499473	498494,1
1347	17,44	17,31	17,37	3,510097	3,551532	3,532388	491373,8	493466,9	492499,7
1357	17,75	17,69	17,69	3,411965	3,430882	3,430882	486418,4	487373,6	487373,6
1367	18	18,06	18	3,333561	3,314846	3,333561	482458,5	481513	482458,5
1377	18,31	18,37	18,31	3,237309	3,218809	3,237309	477593,1	476657	477593,1
1387	18,62	18,69	18,62	3,142195	3,120882	3,142195	472776,6	471695,8	472776,6

1397	18,87	19,06	18,94	3,066363	3,009274	3,045275	468927,8	466023,7	467855,8
1407	19,19	19,37	19,25	2,97049	2,917172	2,952668	464047	461323,9	463137,6
1418	19,44	19,75	19,62	2,896559	2,806109	2,843871	460269,3	455627,6	457568,4
1428	20,12	20,5	20,25	2,699997	2,593184	2,663205	450148,7	444590,3	448239,4
1438	21,06	21,5	21	2,439936	2,323129	2,456113	436523,9	430288,8	437381,1
1448	21,87	22,31	21,69	2,22743	2,116692	2,273695	425114,6	419042,4	427623,9
1458	22,37	23	22,12	2,101853	1,949882	2,164098	418221,2	409694,4	421653,8
1468	22,69	23,12	22,44	2,023782	1,921736	2,084621	413868,1	408090	417265
1478	23,12	23,62	22,94	1,921736	1,807246	1,964052	408090	401472,7	410498,9
1488	23,56	24,25	23,56	1,820747	1,669416	1,820747	402261,1	393287,4	402261,1
1498	24,12	25,12	24,19	1,697269	1,490896	1,682233	394962,6	382257,5	394059,7
1508	24,81	25,87	24,75	1,552937	1,347942	1,565145	386151,7	372997,6	386910
1518	25,44	26,31	25,37	1,42867	1,268736	1,442125	378278,8	367669,8	379145,6
1528	25,62	26,25	25,69	1,394475	1,279336	1,381334	376059	368391,8	375199,3
1538	25,87	26,25	26,06	1,347942	1,279336	1,31332	372997,6	368391,8	370687,6
1548	26,31	27,25	26,44	1,268736	1,110848	1,245987	367669,8	356541,4	366110,3
1558	26,75	27,69	26,87	1,192929	1,04213	1,172838	362418,2	351448,7	360999
1568	27,25	28,37	27,31	1,110848	0,942244	1,101285	356541,4	343720,9	355842,7

1578	27,94	28,94	27,87	1,004525	0,864256	1,01495	348587,7	337374,3	349386,4
1588	28,44	29,31	28,44	0,932388	0,81635	0,932388	342935,1	333317,4	342935,1
1599	28,94	30	28,81	0,864256	0,732535	0,881591	337374,3	325881,7	338811,4
1609	29,06	30,06	29,12	0,848488	0,725578	0,840687	336053,2	325243	335394,5
1619	29,31	30,69	29,75	0,81635	0,655627	0,762087	333317,4	318611,9	328556,5
1629	29,87	31,5	30,37	0,747788	0,573685	0,690457	327269,9	310284,4	321963
1639	30,81	32,56	31,12	0,642931	0,479132	0,611038	317364,2	299714,7	314163,7
1649	31,69	33,63	31,94	0,55571	0,397017	0,532755	308362,8	289410,3	305852,5
1659	32,5	34,38	32,75	0,48412	0,346714	0,463612	300303,2	282399,5	297858,5
1669	33,25	35,19	33,13	0,424732	0,298493	0,433808	293028,6	275018,6	294180,6
1679	33,5	34,81	33,25	0,406325	0,32036	0,424732	290643,1	278456,9	293028,6
1689	32,94	34	32,56	0,448507	0,371491	0,479132	296013,8	285930,2	299714,7
1699	31,75	32,38	31,31	0,550129	0,494225	0,592125	307758,5	301483,8	312218
1709	30,94	31	30,31	0,629399	0,62323	0,697148	316018,1	315398,8	322595,3
1719	30,19	30,06	29,62	0,710683	0,725578	0,777818	323863,5	325243	329956
1729	29,75	29,56	29,25	0,762087	0,785163	0,823976	328556,5	330603,9	333972
1739	29,69	29,5	29,25	0,769316	0,792562	0,823976	329201,6	331253,1	333972
1749	29,81	29,75	29,69	0,754911	0,762087	0,769316	327912,5	328556,5	329201,6

1759	30,25	31,06	30,62	0,70389	0,61711	0,663124	323228,8	314780,6	319342
1770	30,94	33,25	31,94	0,629399	0,424732	0,532755	316018,1	293028,6	305852,5
1780	32,31	35,56	33,5	0,500198	0,278426	0,406325	302174,6	271711,5	290643,1
1790	33,06	36,88	34,38	0,439177	0,215901	0,346714	294854,7	260234	282399,5
1800	33,5	37,38	34,88	0,406325	0,195587	0,316233	290643,1	256014,2	277820,3
1810	33,38	37,75	35,13	0,415076	0,181637	0,301859	291785,7	252935,6	275558,6
1820	33,56	36,88	35,31	0,402007	0,215901	0,291855	290073,5	260234	273941,6
1830	33,69	37,5	35,06	0,392782	0,190965	0,305827	288843,1	255011,6	276190
1840	33,31	36,19	34,25	0,420252	0,246891	0,35503	292454,3	266171,8	283602,5
1850	32,44	34,13	32,94	0,489151	0,362853	0,448507	300892,9	284717,4	296013,8
1860	31,62	32,44	31,62	0,562279	0,489151	0,562279	309069,4	300892,9	309069,4
1870	30,81	31,19	30,75	0,642931	0,604014	0,649254	317364,2	313445,5	317987,5
1880	30,12	30,19	30	0,718673	0,710683	0,732535	324605,6	323863,5	325881,7
1890	29,62	29,44	29,44	0,777818	0,800015	0,800015	329956	331903,6	331903,6
1900	29,19	28,94	29	0,831657	0,864256	0,856344	334627,8	337374,3	336713,1
1910	28,81	28,44	28,56	0,881591	0,932388	0,915675	338811,4	342935,1	341592,2
1920	28,37	27,94	28,19	0,942244	1,004525	0,967949	343720,9	348587,7	345749,8
1930	28	27,5	27,87	0,995654	1,071404	1,01495	347904,5	353638,9	349386,4

1941	27,56	27,06	27,5	1,062094	1,141534	1,071404	352945,8	358763,3	353638,9
1951	27,06	26,62	27,12	1,141534	1,214976	1,131777	358763,3	363962	358060,2
1961	26,44	26,06	26,69	1,245987	1,31332	1,203068	366110,3	370687,6	363129,9
1971	25,81	25,5	26,25	1,359008	1,417207	1,279336	373730	377537,4	368391,8
1981	25,12	24,94	25,75	1,490896	1,526708	1,370139	382257,5	384513,9	374463,9
1991	24,69	24,44	25,31	1,577419	1,62926	1,453727	387669,8	390851,7	379890,1
2001	24,37	24,06	25	1,643978	1,710227	1,514706	391747,3	395738,2	383760,3
2011	24,25	23,75	24,62	1,669416	1,778216	1,59182	393287,4	399769,8	388558,1
2021	24,06	23,37	24,31	1,710227	1,863928	1,656664	395738,2	404767,8	392516,6
2031	23,81	23,06	23,94	1,764921	1,935777	1,736339	398986,3	408891,4	397294
2041	23,44	22,75	23,62	1,847944	2,009345	1,807246	403842,5	413057	401472,7
2051	22,87	22,37	23,31	1,980664	2,101853	1,8777	411439,5	418221,2	405562,7
2061	22,56	21,81	22,94	2,055281	2,24279	1,964052	415631,1	425949,4	410498,9
2071	22,56	21,56	22,44	2,055281	2,307452	2,084621	415631,1	429445,4	417265
2081	22,44	21,31	22,12	2,084621	2,373173	2,164098	417265	432970,2	421653,8
2091	22,19	20,94	21,81	2,14656	2,472349	2,24279	420689,8	438240	425949,4
2101	22,12	20,75	21,5	2,164098	2,524147	2,323129	421653,8	440971	430288,8
2112	22,06	20,56	21,37	2,179199	2,576525	2,357304	422481,8	443719	432121,6

2122	22	20,5	21,25	2,194362	2,593184	2,389102	423311,4	444590,3	433820,4
2132	21,87	20,5	21,19	2,22743	2,593184	2,40509	425114,6	444590,3	434672,3
2142	21,75	20,31	21,06	2,258211	2,646311	2,439936	426785,8	447360,9	436523,9
2152	21,56	20,12	20,87	2,307452	2,699997	2,491364	429445,4	450148,7	439244,2
2162	21,37	20	20,69	2,357304	2,734188	2,540625	432121,6	451918,4	441836,9
2172	21,19	19,75	20,5	2,40509	2,806109	2,593184	434672,3	455627,6	444590,3
2182	20,94	19,5	20,25	2,472349	2,878945	2,663205	438240	459367,2	448239,4
2192	20,69	19,31	20	2,540625	2,934895	2,734188	441836,9	462229,9	451918,4
2202	20,44	19	19,69	2,6099	3,02725	2,823508	445463,4	466938,8	456522,3
2212	20,19	18,69	19,44	2,680154	3,120882	2,896559	449119,6	471695,8	460269,3
2222	20	18,5	19,25	2,734188	3,178874	2,952668	451918,4	474635,3	463137,6
2232	19,81	18,37	19,12	2,788764	3,218809	2,991345	454734,6	476657	465110,3
2242	19,69	18,25	18,94	2,823508	3,255852	3,045275	456522,3	478530,9	467855,8
2252	19,56	18,12	18,81	2,861382	3,296171	3,08449	458466,9	480569,3	469848,7
2262	19,37	17,94	18,62	2,917172	3,352316	3,142195	461323,9	483406	472776,6
2272	19,25	17,81	18,5	2,952668	3,393087	3,178874	463137,6	485465,1	474635,3
2283	19,06	17,62	18,31	3,009274	3,452998	3,237309	466023,7	488490,4	477593,1
2293	18,94	17,5	18,19	3,045275	3,491028	3,274436	467855,8	490410,8	479470,6

2303	18,75	17,37	18	3,102663	3,532388	3,333561	470771,3	492499,7	482458,5
2313	18,69	17,25	17,94	3,120882	3,57071	3,352316	471695,8	494435,9	483406
2323	18,56	17,12	17,81	3,160512	3,612376	3,393087	473705	496542	485465,1
2333	18,44	17,06	17,69	3,19728	3,631658	3,430882	475567,3	497517,1	487373,6
2343	18,25	16,81	17,5	3,255852	3,71233	3,491028	478530,9	501600,5	490410,8
2353	18,06	16,69	17,37	3,314846	3,751234	3,532388	481513	503572,5	492499,7
2363	17,94	16,5	17,25	3,352316	3,813057	3,57071	483406	506710,6	494435,9
2373	17,75	16,31	17,06	3,411965	3,87514	3,631658	486418,4	509868,3	497517,1
2383	17,69	16,25	17	3,430882	3,894795	3,650971	487373,6	510869,5	498494,1
2393	17,56	16,12	16,87	3,471995	3,93746	3,692921	489449,6	513045,6	500617,5
2403	17,5	16,06	16,75	3,491028	3,957186	3,731768	490410,8	514053,1	502585,6
2413	17,37	15,94	16,62	3,532388	3,996701	3,77398	492499,7	516074	504726,4
2423	17,13	15,88	16,56	3,609166	4,016489	3,793505	496379,7	517087,5	505717,5
2433	17,25	15,75	16,5	3,57071	4,059426	3,813057	494435,9	519290	506710,6
2443	17,19	15,75	16,44	3,589922	4,059426	3,832635	495406,8	519290	507705,7
2454	17,06	15,69	16,37	3,631658	4,079271	3,855508	497517,1	520309,8	508869
2464	17	15,63	16,31	3,650971	4,099133	3,87514	498494,1	521331,5	509868,3
2474	16,94	15,56	16,25	3,670315	4,122326	3,894795	499473	522526,1	510869,5

2484	16,87	15,5	16,19	3,692921	4,142222	3,914473	500617,5	523552,2	511872,7
2494	16,81	15,38	16,06	3,71233	4,182057	3,957186	501600,5	525610,5	514053,1
2504	16,69	15,31	15,94	3,751234	4,205319	3,996701	503572,5	526814,8	516074
2514	16,56	15,19	15,81	3,793505	4,245235	4,039598	505717,5	528885,9	518272,3

APPENDIX II

INPUT VALUES FOR CALCULATING PAVEMENT DEFLECTION CONSIDERING TEMPERATURE AND AXLE LOAD

Resilient Strain in HMA	Axle Load	Pavement Temperature	Axle Load Constant	Temperature Constant	Deflection of Pavement
K1	N (kN)	T (Degrees)	K2	K3	Δ_{HMA} (inches)
0,00000007	1000	23,87	0,4791	1,5606	0,00027
0,00000007	1500	24,62	0,4791	1,5606	0,00035
0,00000007	2000	25,06	0,4791	1,5606	0,00041
0,00000007	2500	24,94	0,4791	1,5606	0,00045
0,00000007	3000	25,12	0,4791	1,5606	0,00050
0,00000007	3500	25,37	0,4791	1,5606	0,00054
0,00000007	4000	25,75	0,4791	1,5606	0,00059
0,00000007	4500	26,06	0,4791	1,5606	0,00064
0,00000007	5000	26,12	0,4791	1,5606	0,00067
0,00000007	5500	26,19	0,4791	1,5606	0,00071
0,00000007	6000	26,37	0,4791	1,5606	0,00075
0,00000007	1000	23,87	0,4791	1,5606	0,00027
0,00000007	1500	24,62	0,4791	1,5606	0,00035

0,00000007	2000	25,06	0,4791	1,5606	0,00041
0,00000007	2500	24,94	0,4791	1,5606	0,00045
0,00000007	3000	25,12	0,4791	1,5606	0,00050
0,00000007	3500	25,37	0,4791	1,5606	0,00054
0,00000007	4000	25,75	0,4791	1,5606	0,00059
0,00000007	4500	26,06	0,4791	1,5606	0,00064
0,00000007	5000	26,12	0,4791	1,5606	0,00067
0,00000007	5500	26,19	0,4791	1,5606	0,00071
0,00000007	6000	26,37	0,4791	1,5606	0,00075
0,00000007	1000	23,87	0,4791	1,5606	0,00027
0,00000007	1500	24,62	0,4791	1,5606	0,00035
0,00000007	2000	25,06	0,4791	1,5606	0,00041
0,00000007	2500	24,94	0,4791	1,5606	0,00045
0,00000007	3000	25,12	0,4791	1,5606	0,00050
0,00000007	3500	25,37	0,4791	1,5606	0,00054
0,00000007	4000	25,75	0,4791	1,5606	0,00059
0,00000007	4500	26,06	0,4791	1,5606	0,00064

0,00000007	5000	26,12	0,4791	1,5606	0,00067
0,00000007	5500	26,19	0,4791	1,5606	0,00071
0,00000007	6000	26,37	0,4791	1,5606	0,00075
0,00000007	6500	26,69	0,4791	1,5606	0,00079
0,00000007	7000	26,94	0,4791	1,5606	0,00083
0,00000007	7500	27,12	0,4791	1,5606	0,00087
0,00000007	8000	27,12	0,4791	1,5606	0,00090
0,00000007	8500	27,12	0,4791	1,5606	0,00092
0,00000007	9000	27,06	0,4791	1,5606	0,00094
0,00000007	10000	27,25	0,4791	1,5606	0,00100
0,00000007	10500	27,50	0,4791	1,5606	0,00104
0,00000007	11000	27,75	0,4791	1,5606	0,00108
0,00000007	11500	28,19	0,4791	1,5606	0,00113
0,00000007	12000	28,62	0,4791	1,5606	0,00118
0,00000007	12500	28,56	0,4791	1,5606	0,00120
0,00000007	13000	28,81	0,4791	1,5606	0,00124

APPENDIX III: INPUT VALUES FOR FIELD CALIBRATION CONSTANT KZ

Kz	C1	C2	K1r	K2r	K3r
1	-57,5815	-12,1605	-3,35412	0,4791	1,5606
-1,28444E-09	-57,5815	-12,1605	-3,35412	0,4791	1,5606
-1,28444E-09	-57,5815	-12,1605	-3,35412	0,4791	1,5606
-1,28444E-09	-57,5815	-12,1605	-3,35412	0,4791	1,5606
-1,28444E-09	-57,5815	-12,1605	-3,35412	0,4791	1,5606
-1,28444E-09	-57,5815	-12,1605	-3,35412	0,4791	1,5606
-1,28444E-09	-57,5815	-12,1605	-3,35412	0,4791	1,5606
-1,28444E-09	-57,5815	-12,1605	-3,35412	0,4791	1,5606
-1,28444E-09	-57,5815	-12,1605	-3,35412	0,4791	1,5606
-1,28444E-09	-57,5815	-12,1605	-3,35412	0,4791	1,5606
-1,28444E-09	-57,5815	-12,1605	-3,35412	0,4791	1,5606

APPENDIX IV: PROCEDURE FOR INSTRUMENTATION AND INSTALLATION

Road IOT Instrumentation Notes

1. Installation

- Pull the two wires (power and antenna) through the PVC pipe.
- Before connecting the **20mm** male and female PVC pipes, apply the provided PVC Weld thoroughly. Connect the male and female.
- Place the device and pipes in the ground.
- Connect the power cable to the solar panel.
- Take down the current GPS location of the device as well as the device ID for the software developer to implement. This will ensure that the location of each device is known should the GPS fail.

2. SigFox Backend

The device transmits the reading to the SigFox backend. This information can be seen at:

https://backend.sigfox.com/

Username: bskrtell@gmail.com

Password: banksZA111/

3. Data package

The data will be sent to the backend as follow:

Bytes	Example	Description
RAW DATA	0f5444330f9313750c2810d6	
BYTE 0,1,2 and 3	0F 54 44 33	***Latitude**
BYTE 4,5,6 and 7	0F 93 13 75	***Longitude**
BYTE 8	0C	**Temperature (degree Celsius)**
BYTE 9	28	**Humidity (%)**
BYTE 10 and 11	10 D6	**Battery Voltage (Milli-Volts)**

* Latitude data should be divided by 10 000 000 to get the correct floating number
* Longitude data should be divided by 10 000 000 to get the correct floating number

C++ RUNTIME SIMULATION PROGRAM

```
//************************************************************
//This is a program to calculate
//the deflection of subgrade due to the load
//acting on a pavement at varying subgrade water content
//************************************************************
//
//NAME: Abejide, Olugbenga Samuel
//REGN:
//************************************************************

#include <iostream>
#include <cmath>
using namespace std;

int main()
{
       float E1, W, G, Y, E2, H, Mr, MR, P, DR1, DR2, R = 1.8;
       float a, b;
       float NF, T, c, d;


       // Input parameters of the resilient modulus of subgrade.
       //Value of water content.
       cout << "Enter the value of the water content, Wc: ";
       cin >> W;
       cout << '\n';

       //Value of ground water table.
       cout << "Enter the value of the ground water table, GWT: ";
       cin >> G;
       cout << '\n';


       //Input value of temperature
       cout << "Enter value of pavement temperature, Tc: ";
       cin >> T;
       cout << '\n';

       cout << "Enter the thickness of soil: ";
       cin >> H;
       cout << '\n';

       cout << "Enter the value of applied load: ";
       cin >> P;
       cout << '\n';


       //Value of stress in pavement in MPa.
       Y = 0.67; //MPa

       //Calculation procedure for the resilient modulus of subgrade

       cout << "*******THE FOLLOWING ARE RESULTS AS COMPUTED IN THE PROGRAM******" <<
       endl;
```

```
        cout << '\n';
        cout << '\n';

        //cout << "Resilient modulus of subgrade, Er = " << E1 << endl;
        cout << '\n';


        a = pow(G, 0.1192);
        b = ((51.72 * (1 / (0.3586 * a))) / W);
        E1 = 2555 * pow(b, 0.64);
        cout << "Resilient modulus of subgrade, Er = " << E1 << endl;
        cout << '\n';

        //Resilient strain is the ration of stress to strain
        //The calculation for resilient strain is thus.
        E2 = Y / E1;
        cout << "Resilient strain, Esg = " << E2 << endl;
        cout << '\n';

        //*************************************************************
        //***Input parameters for local repetition to failure.


        c = 2.16 * pow(10, -14) * pow(1.16, T);
        d = pow((E2 * pow(10, -6)), 4.2);
        NF = c / d;
        cout << '\n';
        cout << "Load repetition to failure, Nf = " << NF << endl;
        cout << '\n';


        // Mr(subgrade) = Er
        Mr = E1;
        MR = 0.2 * pow(H, 0.45) * Mr; //MR is for subbase; Mr is for subgrade

        cout << "MR (strain of sub base) = " << MR << endl;
        cout << '\n';


        DR1 = (0.24 * P) / (MR * R); // Deflection of sub base
        DR2= (0.24 * P) / (Mr * R); //Deflection of subgrade
        cout << "Deflection of subgrade = " << DR2 << endl;
        cout << '\n';
        cout << "Deflection of sub base = " << DR1 << endl;
        cout << '\n';


        return 0;
}
```
